# Carboxymethyl Polysaccharides/Montmorillonite Biocomposite Films and Their Sorption Properties

**DOI:** 10.3390/polym17152130

**Published:** 2025-08-01

**Authors:** Adrian Krzysztof Antosik, Marcin Bartkowiak, Magdalena Zdanowicz, Katarzyna Wilpiszewska

**Affiliations:** 1 Department of Organic Chemical Technology and Polymer Materials, Faculty of Chemical Technology and Engineering, West Pomeranian University of Technology in Szczecin, Piastów Ave. 42, 71-065 Szczecin, Poland; marcin.bartkowiak@zut.edu.pl (M.B.); katarzyna.wilpiszewska@zut.edu.pl (K.W.); 2 Center of Bioimmobilisation and Innovative Packaging Materials, Faculty of Food Sciences and Fisheries, West Pomeranian University of Technology in Szczecin, Janickiego 35, 71-270 Szczecin, Poland; magdalena.zdanowicz@zut.edu.pl

**Keywords:** hydrogel, carboxymethyl cellulose, carboxymethyl starch, montmorillonite, sorption

## Abstract

The production of bionanocomposite films based on carboxymethyl derivatives of starch and cellulose with sodium montmorillonite (MMT-Na) as a filler was described. The developed films with high absorbency can be used in the preparation of adhesive dressings for wounds oozing as a result of abrasions or tattoos. Carboxymethyl cellulose (CMC), carboxymethyl starch (CMS), and potato starch were used as the raw materials for film manufacturing. Citric acid was used as a crosslinking agent and glycerol as a plasticizer. The following parameters were evaluated for the obtained films: solubility in water, swelling behavior, moisture absorption, and mechanical durability (tensile strength, elongation at break, and Young’s modulus). This study revealed that filler concentration has a significant influence on the stability, durability, and moisture absorption parameters of films. The best nanocomposite with a high absorption capacity was a two-component film CMS/CMC containing 5 pph of sodium montmorillonite and can be used as a base material for wound dressing, among other applications.

## 1. Introduction

The demand for polymer materials has been growing for years, and this process shows no signs of decreasing. For over two decades, scientists have successfully started to introduce biopolymers from renewable sources into use, replacing petroleum-based materials that, despite their good utility properties, are non-renewable materials that will eventually run out. One of the groups of green renewable materials that has attracted interest is polysaccharides, especially starch, cellulose, and their carboxymethyl derivatives [1].

Starch (S) is a white, semi-crystalline substance without taste or smell and is insoluble in cold water, but in hot water, it forms a paste. Starch hydrolyzes only to α-D-glucose. Chemically, it is not a uniform material, because it actually consists of two fractions, amylose and amylopectin [2,3]. It is primarily used in the food, paper, and pharmaceutical industries. Due to its polymeric form, it has been used in the textile and cosmetics industries and for the production of adhesives (especially pastes) [4,5,6]. It is a film-forming material characterized by high values of mechanical properties and relatively low moisture absorption [7,8,9].

Carboxymethyl starch (CMS) is an anionic starch derivative with great importance in the pharmaceutical, medicine, cosmetics, and food industries, environmental protection, and many other industrial applications [10]. CMS is soluble in cold water, non-toxic, biodegradable, and biocompatible with the human body [11]. In this work, the method of preparing hydrophilic films based on a polysaccharide-based system by casting from an aqueous solution was described. Citric acid (CA) was used as a crosslinking agent because of its multi-carboxyl structure, able to crosslink carboxymethyl polysaccharides and starch [12,13,14,15,16,17,18,19,20]. The effect of citric acid content on the physicochemical properties of the obtained films was evaluated, and lower CA concentrations improve tensile strength and barrier properties, while higher concentrations decrease strength and increase water permeability [13]. Carboxymethyl starch-based films with enhanced hydrophilicity could be used in agriculture, e.g., for seed tape production. The main roles of such tapes are improving germination and seed protection, and retaining humidity [12]. The use of biodegradable and green raw materials was an additional benefit of the obtained films. These materials have gained a lot of interest in the scientific world, and consequently, many applications have been developed [21,22,23].

There are many reports on the formulation of biodegradable films based on polysaccharides; their derivatives, i.e., starch (S) [24,25], carboxymethyl starch [12,26], and carboxymethyl cellulose (CMC) [27,28]; or blends of starch with other polysaccharides such as chitosan, pectin, carrageenan, hemicellulose, or alginate [17,29]. The physicochemical properties (tensile strength, moisture absorption, and solubility in water) of such films depend greatly on their composition [7,30,31]. For instance, introducing carboxymethyl cellulose to a starch matrix resulted in a slight reduction in tensile strength and solubility in water, whereas elongation increased [32,33,34].

Montmorillonite was discovered in the mid-nineteenth century in French Montmorillon. It is a mineral clay of volcanic origin and is a component of bentonite rock. It is a layered silicate with a lamellar structure and a density of 1.9–2.7 g/cm^3^. Montmorillonite is strongly hydrophilic. Small amounts of aluminosilicate incorporated within the polymer matrix cause an increase in Young’s modulus, improve the mechanical properties of the composite, reduce gas permeability, and improve heat resistance and flame retardance [35]. The addition of montmorillonite (MMT, 7 parts by weight) into starch/CMC films improved the tensile strength of the film from 9.83 to 27.55 MPa. In addition, it resulted in a decrease in water solubility and moisture adsorption from ca. 18 to 16% and ca. 24 to 14%, respectively [20].

The main goal of this research was to obtain polysaccharide-based films that could be used for adhesive wound dressings. The films were prepared using i) carboxymethyl derivatives of starch and cellulose, and ii) a blend of carboxymethyl derivatives of starch and cellulose with starch. The effect of MMT content on the mechanical and sorption properties of the film composites was evaluated. The system exhibiting the most promising properties was selected for the preparation of a wound dressing patch prototype.

**Figure 1:**
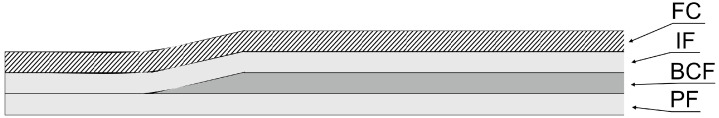
Scheme of proposed dressing.

The new bio-based patch could be used to dress abrasions, wounds with exudate, or tattooed skin; the layer of plasma-permeable film does not adhere to the site of the exudate, and the biodegradable film in the bag will draw in the exudate, separate it from the body, and bind it, potentially accelerating the healing process. The design of the dressing is shown in Figure 1. It consists of a permeable layer (PF), an absorbent biocomposite layer (BCF), an impermeable layer (IF), and a fabric coating layer (FC) protecting the dressing from mechanical damage.

## 2. Materials and Methods

### 2.1. Materials

Potato starch (S) was obtained from Nowamyl S.A. (Łobez, Poland). Carboxymethyl cellulose (degree of substitution-DS: 0.7, Pollocel AS-2/90) was purchased from Pronicel Sp. o.o. (Radom, Poland). Glycerol (p.a.), and monohydrate citric acid (CA) was purchased from Chempur (Piekary Śląskie, Poland). Carboxymethyl starch with DS = 0.7 was prepared according to the method described in [10]. Sodium montmorillonite Na^+^ (MMT-Na) was isolated from Bentonit special PLUS (Zębiec, Starachowice, Poland).

### 2.2. Isolation of Sodium Montmorillonite

**Figure 2:**
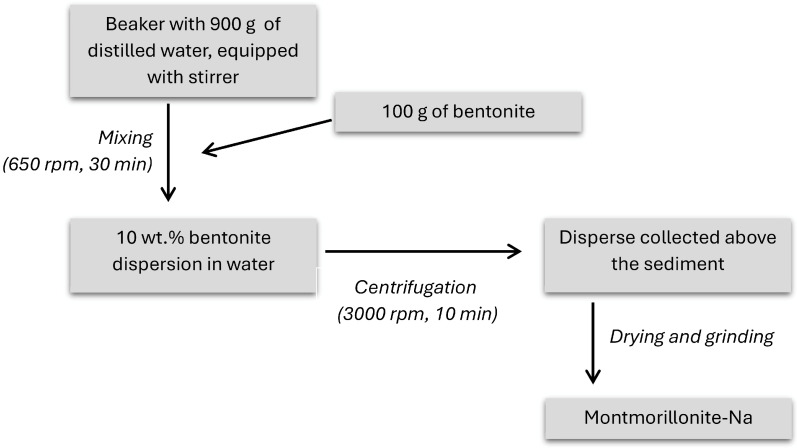
Scheme of montmorillonite isolation from the bentonite clay.

To the 2000 cm^3^ beaker containing 900 g of distilled water, 100 g of bentonite was added and stirred for 30 min to create a homogeneous mixture. Subsequently, 10 wt% aqueous dispersion of bentonite was centrifuged (Sigma 6K15 centrifuge, Osterode am Harz, Germany) for 10 min at 3000 rpm. The purified MMT dispersion above the sludge was removed, dried, and ground. Figure 2 presents the scheme of montmorillonite isolation from the bentonite source [36].

### 2.3. Preparation of Polysaccharide-Based Films

The CMS/CMC film with sodium montmorillonite was prepared as follows: the aqueous dispersion (100 mL) of montmorillonite (0, 1, 3, 5, and 7 pph on a basis of total dry polysaccharide derivatives weight), 2 g of glycerol, and 2 g of citric acid were prepared and stirred for 30 min at room temperature until well dispersed. Subsequently, a polysaccharide mixture (CMS/CMC wt. ratio 1:1—3 g was added and stirred until homogeneous. Then, the system was poured into PTFE molds and placed in a dryer for 48 h at 60 °C. The obtained films (thickness: 100–200 μm) were removed from the mold and tested.

The CMS/CMC/S films were prepared according to previous work [37,38] with MMT addition as described above. The polysaccharide CMS/CMC/S wt ratio was 1:1:2.

### 2.4. Methods

#### 2.4.1. Solubility in Water

The solubility in water test was performed as follows: Three samples (1.5 cm × 1.5 cm) of each film were placed in a dryer to a constant mass. The dried samples were weighed and placed in vials, which were filled with 50 mL of distilled water. After 24 h, the samples were removed and dried to a constant mass (ca. 24 h) at 60 °C. The dry samples were weighed again. The solubility in water was calculated using the following Formula (1) [20,39]:

(1) $$TSM=\frac{M_{1}-M_{2}}{M_{1}}*100\%$$

where

*TSM* (Total Soluble Matter)—solubility in water [%];

*M*_1_—mass of the dry sample [g];

*M*_2_—mass of the sample after soaking and drying [g].

#### 2.4.2. Swelling in Water

For the swelling in water test, five samples (2.5 cm × 2.5 cm) of each film were placed in a dryer to remove moisture (to a constant mass). The dried samples were weighed and placed in vials, which were filled with 50 mL of distilled water. After 24 h, the samples were removed and wiped off with lint-free paper. Then, the mass of the sample was measured. The swelling in water values was calculated using the following Formula (2) [23]:

(2) $$S_{m}=\frac{M_{2}-M_{1}}{M_{2}}*100\%$$

where

*S_m_* (mass swelling degree)—swelling in water [%];

*M*_1_—mass of the dry sample [g];

*M*_2_—mass of the sample after soaking [g].

Moreover, the surface swelling degree (swelling dimension expansion) of composite films was measured as the change in the film surface area after 24 h of immersion in distilled water, according to Formula (3). The initial surface area of the dried samples was 6.25 cm^2^.

(3) $$S_{s}=\frac{S_{2}-S_{1}}{S_{2}}*100\%$$

where

*Ss*—surface swelling degree [%];

*S*_1_—surface of the dry sample [cm^2^];

*S*_2_—surface of the swelled sample after 24 h immersion [cm^2^].

The swollen biocomposite is relatively soft, and measuring the thickness of the film under pressure was challenging. Thus, measuring the volume swelling degree was not considered.

#### 2.4.3. Moisture Absorption

To evaluate the moisture absorption, five (1.5 cm × 1.5 cm) samples of each variant were placed in a dryer to dry (to a constant mass). Then, they were weighed and placed in a climate chamber (humidity 55 ± 2%, temperature 25 ± 2 °C). The samples were weighed 3, 5, 7, 24, 48, and 72 h after being placed in the climatic chamber. The moisture absorption was calculated using Formula (4):

(4) $$A_{t}=\frac{M_{t}-M_{0}}{M_{t}}*100\%$$

where

*A_t_*—moisture absorption after time t [%];

*M*_0_—mass of the dry sample [g];

*M_t_*—sample mass after t time: 3, 5, 7, 24, 48, and 72 h [g].

#### 2.4.4. DMTA

The dynamic mechanical thermal analyses (DMTAs) of the CMS/CMC films were performed using DMA Q800 (TA Instruments, New Castle, DE, USA). The measurements were carried out with a film tension clamp at a frequency of 1 Hz, heating rate of 3 °C/min, and temperature range from 0 to 100 °C.

#### 2.4.5. XRD

The degree of montmorillonite dispersion in modified polysaccharide matrix was investigated with X-ray diffraction analysis (diffractometer X’pertPro, PANalytical (Malvern Panalytical, Malvern, UK) operated at the Co Kα wavelength 1514 Ȧ). The composite films were dried and ground before measurement.

#### 2.4.6. FTIR

FTIR analyses of obtained CMC/CMS films were carried out using a spectrometer Nexus Thermo Nicolet (Thermo Fisher Scientific Inc., Waltham, MA, USA), equipped with an ATR attachment and OMNIC 9 Software. Samples were washed with distilled water to remove CA residues and dried at 50 °C [40].

#### 2.4.7. TEM

TEM analyses of CMS/CMC films containing MMT-Na were performed using a transmission electron microscope, Jeol JEM 1200 EX (Jeol Co., Ltd., Tokyo, Japan).

#### 2.4.8. Mechanical Properties

The tensile tests of the biocomposite films were performed using the INSTRON testing machine (Instron 4026, Instron Co., Ltd., Norwood, MA, USA), according to the ISO 527-3:2018 standard [41]. The initial sample was 50 mm long, 10 mm wide, and about 0.1–0.2 mm thick. During the tests, the following parameters were measured: elongation at break, tensile strength, and Young’s modulus. The speed of the mobile clamp was 10 mm/min. Seven repetitions of each material type were tested [42,43].

## 3. Results and Discussion

### 3.1. Solubility in Water

**Figure 3:**
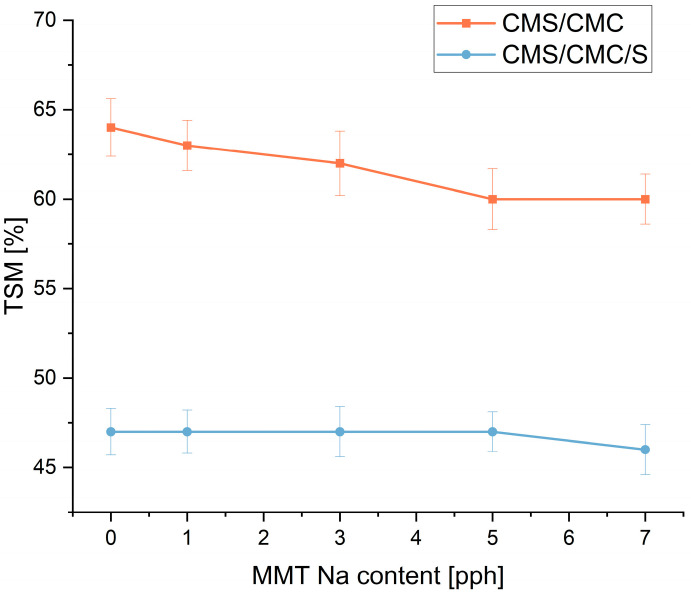
Solubility in water of polysaccharide and biocomposite films as a function of filler content.

The effect of MMT-Na content on the biocomposite swelling in water is presented in Figure 3.

Generally, with an increase in montmorillonite content, the solubility in water decreased. However, for CMS/CMC two-component films, the decrease was more significant and almost linear, dropping from 64% to 60% for an MMT-Na content of 5 pph and higher. Such an effect was also reported for CMS/CMC/S three-component systems [20,44]. In the case of anionic biopolymers, the drop in the TSM value can be caused by the interaction between the OH groups of the clay platelets and the carbonyl groups of polymer chains [45] forming a more “packed” 3D structure. Moreover, highly hydrophilic carboxymethyl polysaccharidic chains and starch can intercalate into the MMT gallery [20,46], being trapped in the composite and leading to reduced interaction with water.

For CMS/CMC/S films, the solubility in water only slightly decreased, and generally, these series exhibited lower TSM, which might be caused by the presence of thermoplastic starch that is less hydrophilic than its carboxymethylated derivative.

### 3.2. The Swelling Effect

**Figure 4:**
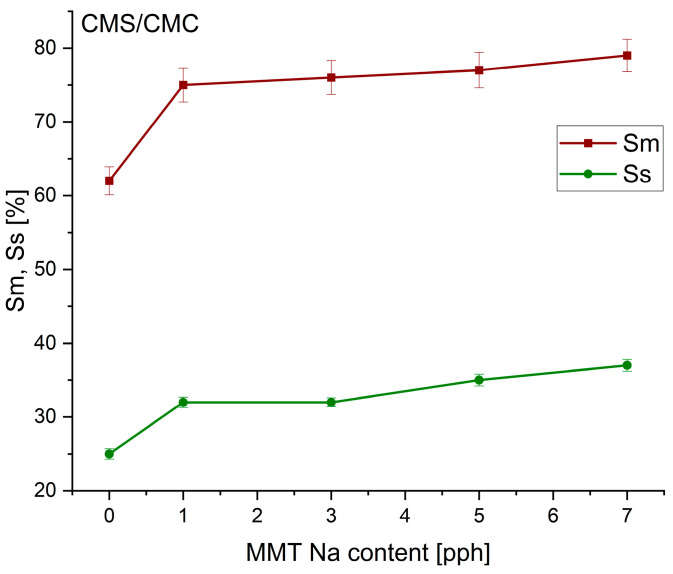
Influence of MMT content on swelling in water (S_m_) and surface swelling ratio (S_s_) of CMS/CMC/MMT-Na biocomposites.

**Figure 5:**
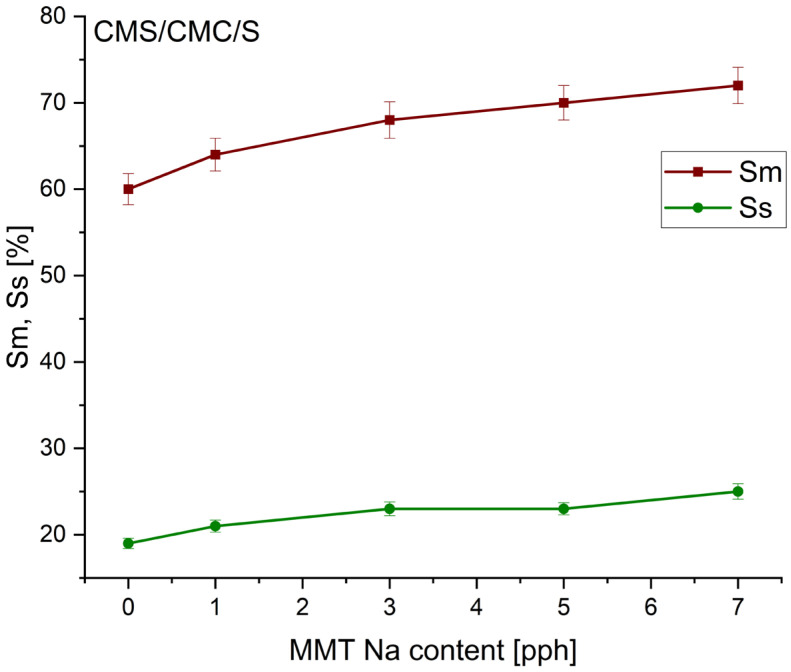
Influence of MMT content on swelling in water (S_m_) and surface swelling ratio (S_s_) of CMS/CMC/S/MMT-Na biocomposites.

The swelling in water and surface swelling degree of the biocomposite films were presented in Figure 4 and Figure 5.

With an increase in MMT-Na content, the swelling in water significantly increased (about 20% for CMS/CMC with 7 pph MMT, and 10% for CMS/CMC/S with 7 pph MMT). The surface swelling ratio also increased with the filler content increase. This could be a result of hydrogen bonding between molecules, forming a large framework that is able to collect water [47]. Intercalated polymer chains unfold after water sorption, increasing the framework’s density and Sm and Ss without increasing the material’s solubility (Figure 4 and Figure 5). The swelling parameters are lower for CMS/CMC/S than for CMS/CMC films.

### 3.3. Moisture Absorption

**Figure 6:**
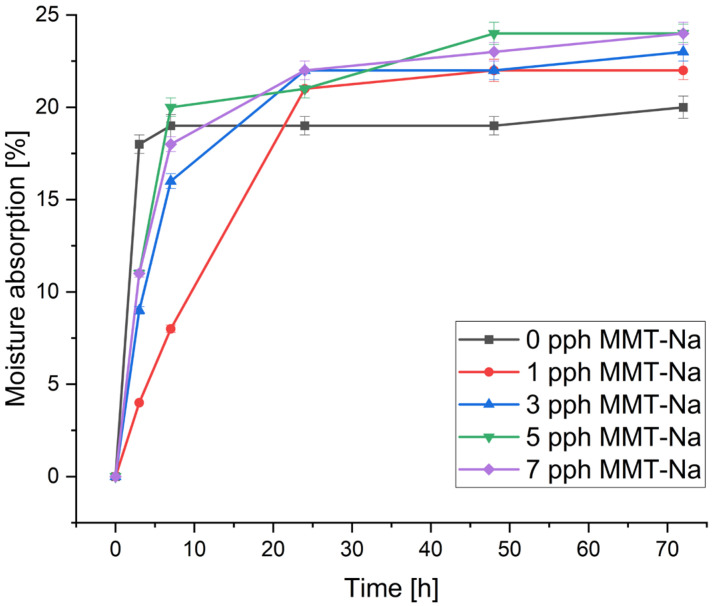
Influence of filler concentration and time on water absorption of CMS/CMC/MMT-Na films.

**Figure 7:**
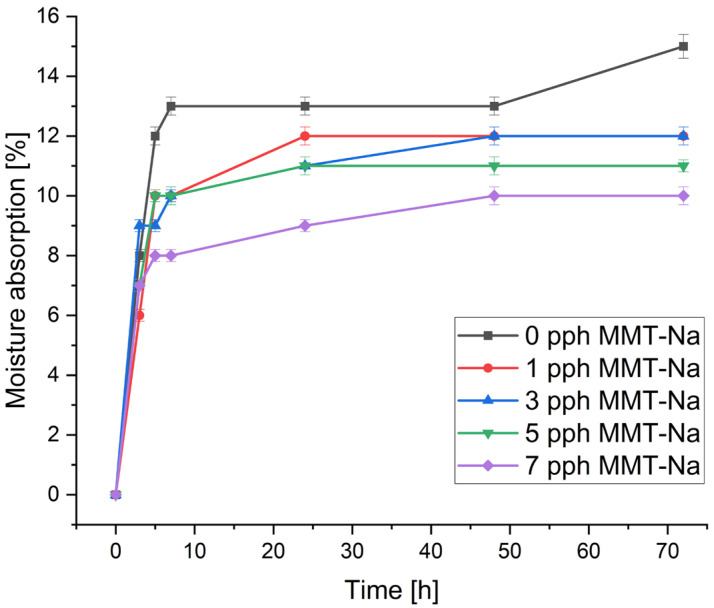
Influence of filler concentration and time on water absorption of CMS/CMC/S/MMT-Na films.

The moisture absorption of the prepared biofilms is presented in Figure 6 and Figure 7. The moisture absorption exhibits a similar trend, characterized by a rapid initial rise and subsequent stabilization within a few hours. The stabilization of CMS/CMC systems occurred after ca. 24 h, and for films containing starch, it occurred after ca. 10 h. Additionally, the latter absorbed less moisture than CMS/CMC films.

Changes in MMT-Na concentration resulted in different effects on water absorption, which referred to the biocomposite matrix composition. In CMS/CMC films, a higher filler content increased moisture absorption, but this trend was inverted for CMS/CMC/S films. This can be caused by the more compact structure formed between clay crystallites and the biopolymer matrix, reduced diffusion of water molecules, and the hydrophilic character of the matrix. Similar results were reported by Almasi et al. [20] for composite films based on starch and CMC films crosslinked with CA and MMT.

### 3.4. DMTA

The Dynamic Mechanical Thermal Analysis was carried out for the obtained films.

**Figure 8:**
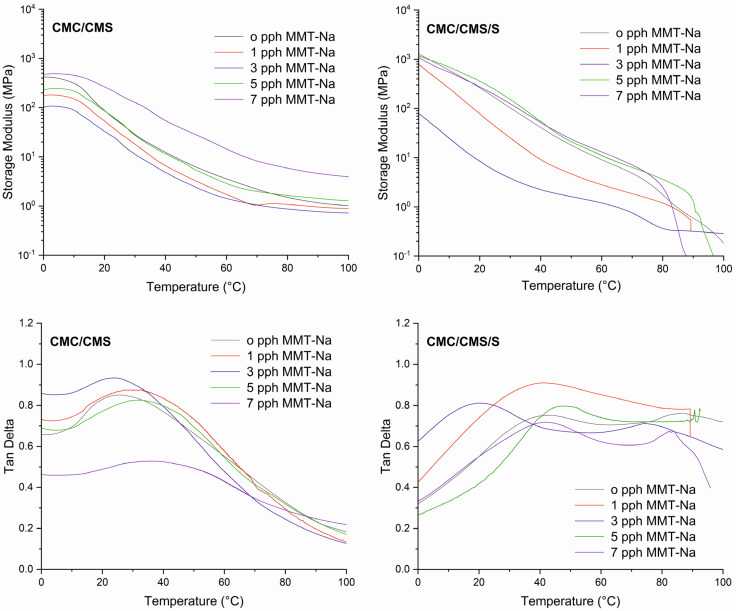
DMTA results (storage moduli and tanδ curves) for CMC/CMS and CMC/CMS/S films with different amounts of sodium montmorillonite (MMT-Na).

Figure 8 shows the storage moduli and tanδ curves for materials based on carboxymethylated polysaccharides with various clay contents. As can be seen, CMC/CMS/S films exhibited a higher storage modulus at low temperatures compared to CMC/CMS films, probably caused by the presence of TPS. The influence of MMT-Na on this parameter is not linear, and only a higher content of the filler (from 5 pph) increased the storage moduli. The lowest modulus was obtained with 3 pph, which corresponds to the mechanical test results (see the tensile strength results and relevant discussion in Section 3.5). There was a sharp drop in moduli at ca. 10–15 °C only for CMC/CMS films, which could be attributed to the glass transition of polymeric chains; however, this drop was not as pronounced for CMC/CMS/S (20–30 °C), except for samples with low clay content. Due to the complex structure (comprising two or three types of amorphous polymers with different chemical structures), H-bonding, the average Mw, the presence of glycerol as a plasticizer and citric acid as a co-plasticizer/crosslinking agent, and the aluminosilicate filler, the change in mobility is very gradual and may be related to moisture evaporation, which acts as a co-plasticizer and shifts the peak towards lower temperatures [48]. In the figure showing tanδ for CMC/CMS in the range of ca. 24–35 °C, α-relaxation peaks are observed, corresponding to the mobility of polymer chains [49]. The addition of the filler, except for the sample with 3 pph of the filler, led to an increase in the maximum α-peak temperature from 24.7 °C for CMC/CMS to 35 °C for CMC/CMS 7 MMT-Na. A similar trend was observed for CMC/CMS/S; however, the range of the peak maxima is higher, at 41.7–46.8 °C. This phenomenon was caused by a restriction of the polymer chains’ mobility by the clay. For CMC/CMS/S, a second peak with a low intensity at a wider range of temperatures can be observed, and this peak is attributed to the α-relaxation of TPS fraction in the materials [50].

### 3.5. Measurement of X-Ray Diffraction Analysis (XRD)

**Figure 9:**
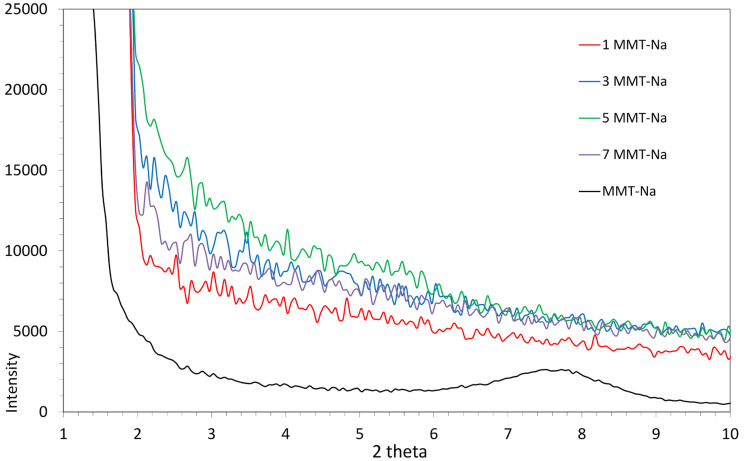
XRD diffractograms of pristine MMT-Na and CMC/CMS/S films with various filler contents.

The degree of montmorillonite dispersion in the modified polysaccharide matrix was investigated with X-ray diffraction analysis. In Figure 9, the sample XRD patterns of the CMC/CMC/S biocomposite with various concentrations of MMT-Na are shown, together with the pristine MMT-Na filler pattern. The clay exhibited a single diffraction peak at 7.8 ° (nm) corresponding to the aluminosilicate platelets’ gallery. In the composite biofilms, this peak flattened, indicating the exfoliation of the filler into the polysaccharidic matrix, independent of the filler content. Similar results were obtained in our previous studies [46].

### 3.6. Fourier Transform Infrared Spectroscopy FTIR

**Figure 10:**
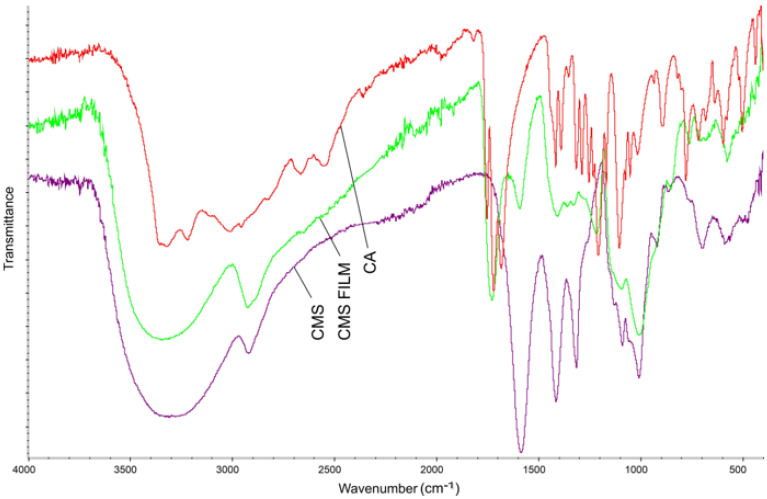
FTIR spectra of CA, CMS, and CMS film crosslinked with citric acid.

**Figure 11:**
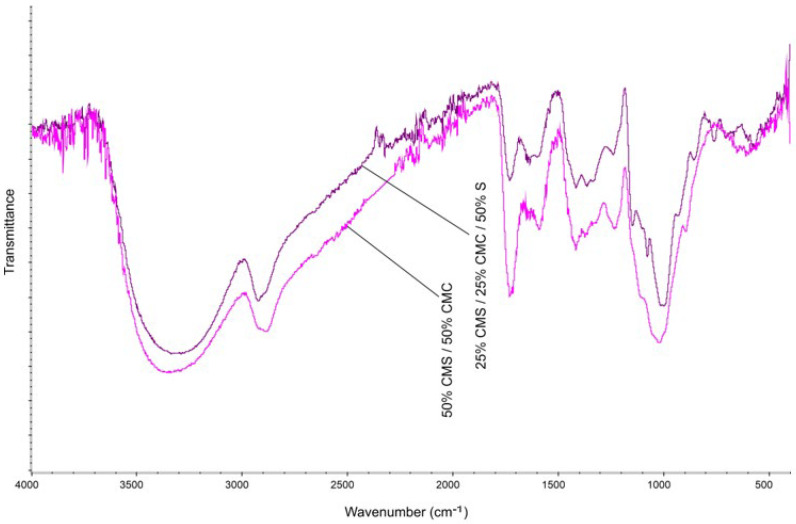
FTIR spectra of CMS/CMC and CMS/CMC/S film.

The FTIR spectra of the obtained CA, CMS, CMS-based film, and CMS/CMC/S film are presented in Figure 10 and Figure 11. The films were washed before analysis to remove residual CA and dried. The C-O band at ca. 1700 cm^−1^ corresponds to the protonated carboxylic groups of CA [51]. -COO- in unmodified CMS gives a strong band at ca. 1600 cm^−1^, where the intensity of this band corresponds to a high DS value [52].

The strong bands at ca. 1425 cm^−1^ and 1315 cm^−1^ correspond, respectively, to CH_2_ and OH vibrations [45]. The absorption bands at 2900 cm^−1^ and 3000–3600 cm^−1^ correspond to CH_2_ stretching vibrations and CMS hydroxyl groups, respectively [52]. For CMS films, the absorption band of the carbonyl group at ca. 1715 cm^−1^ was shown, which confirms chemical linkages between citric acid and starch via ester bonds.

### 3.7. Transmission Electron Microscope Analysis (TEM)

**Figure 12:**
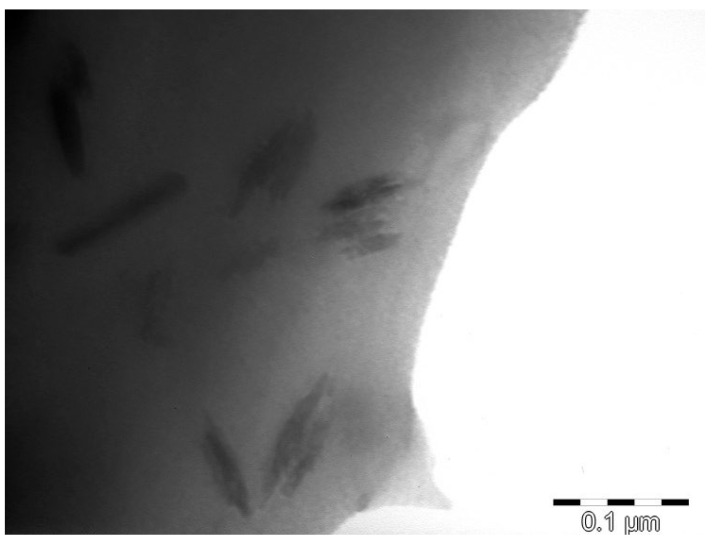
TEM analysis of CMS/CMC/MMT-Na film sample.

TEM analysis of a sample CMS/CMC film containing sodium montmorillonite is presented in Figure 12, where dispersion of exfoliated MMT-Na can be observed.

### 3.8. Mechanical Properties

**Figure 13:**
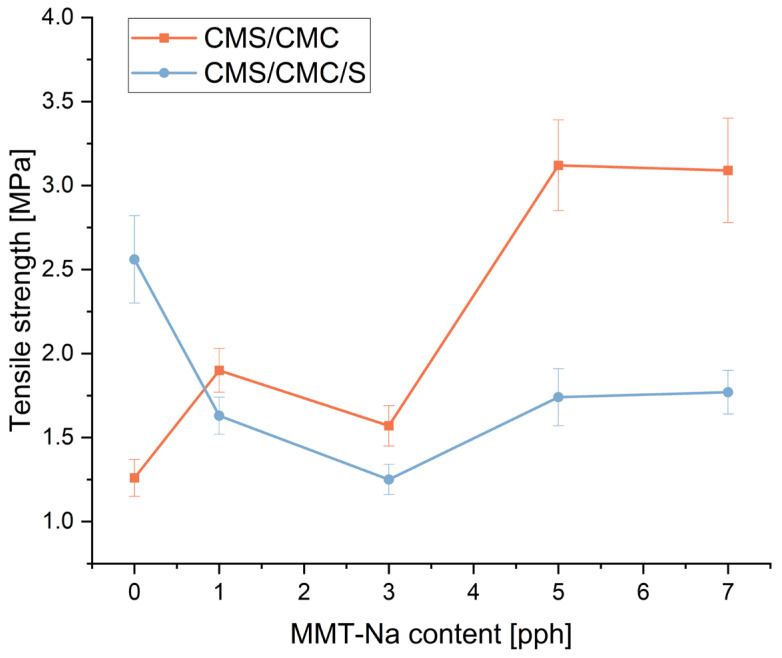
Influence of MMT-Na content on tensile strength of prepared films.

**Figure 14:**
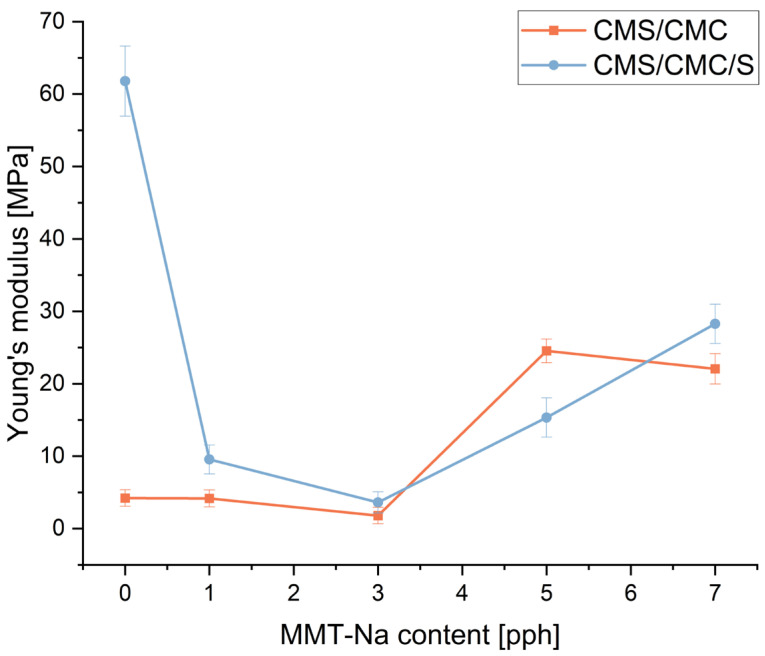
Influence of MMT-Na content on Young’s modulus of prepared films.

**Figure 15:**
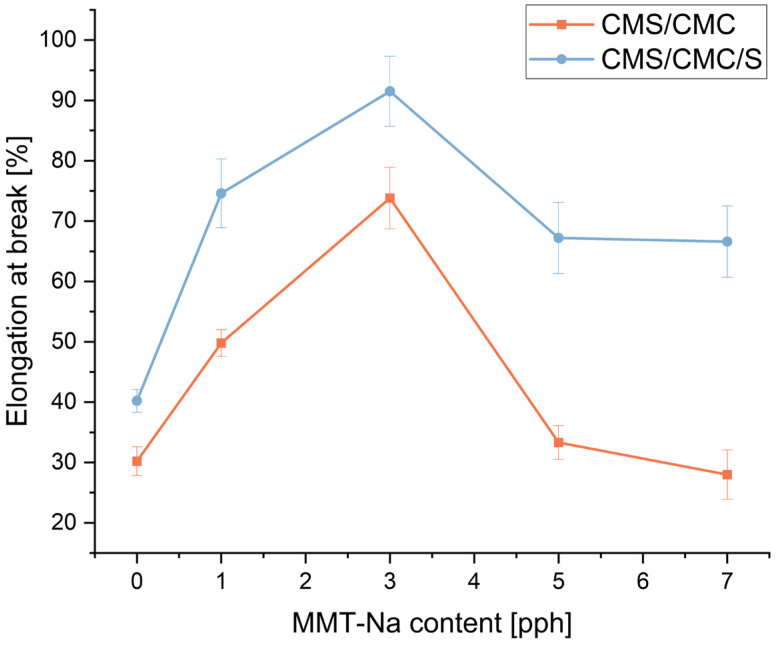
Influence of MMT-Na content on elongation at break of prepared films.

The mechanical performance of the obtained biodegradable films was evaluated by tensile measurements. The results are presented in Figure 13, Figure 14 and Figure 15.

The addition of MMT-Na significantly affects the mechanical performance of the films. Elongation at break increased rapidly after the addition of 1 pph of the filler (three-component film) and 3 pph (two-component film), and then decreased to the starting level (pure sample). Tensile strength and Young’s modulus change differently for both types of films, but the observed trend is similar. With increasing filler concentrations, these values first decrease and then increase gradually. Only the ranges are different. This trend can be caused by two phenomena. The increase in elongation at break and decrease in tensile strength and Young’s modulus for composites with the filler content up to 3 pph can be caused by a restricted crosslinking reaction between CA and polymers, resulting from the presence of clay. Unreacted CA acts as a plasticizer. Similar results were reported by Zdanowicz and Johansson for starch-based films modified with choline citrate and MMT addition [53]. A higher concentration of the filler in the matrix leads to more H-bonds, and a more “dense” structure, overcoming the crosslinking hindrance. CMC/CMC/S films turned out to be more elastic but less durable. CMC/CMS films are more durable with an increase in the filler concentration but more rigid.

Strength tests allowed us to assess the influence of filler content and base composition of films on their mechanical properties. However, it should be remembered that these properties are not the most important from the point of view of the applications assumed in the studies. The absorbent film will ultimately be reinforced with a carrier film, so its ability to absorb moisture is more important.

### 3.9. Wound Dressing Model Preparation

Among the biocomposite films obtained in this study, the most promising performance, referring to the planned application, was exhibited by the film CMS/CMC containing 5 pph of MMT-Na. Reduced solubility in water, increased swelling, and moisture absorption were crucial. Moreover, adding 5 pph of the filler resulted in a significant tensile strength improvement (from 1.3 MPa up to 3.1 MPa), while maintaining the same level of elongation at break as without the filler (ca. 30%).

**Figure 16:**
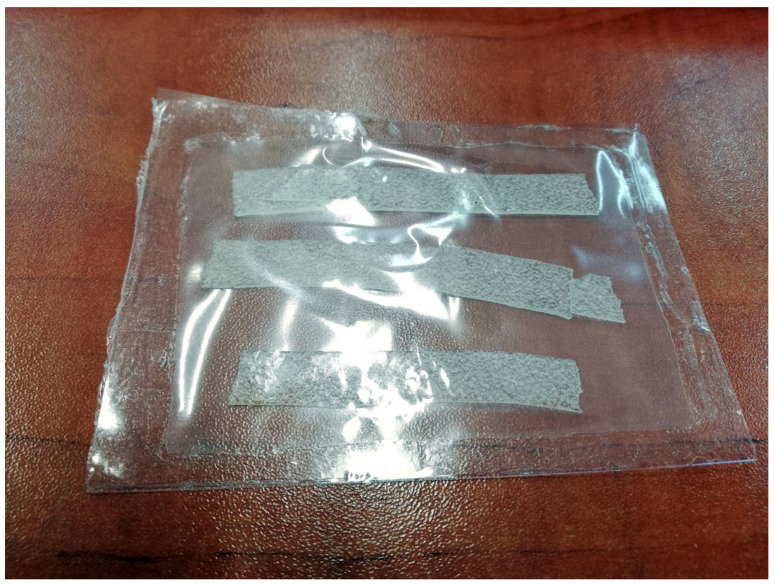
One-sided permeable bag with CMS/CMC-MMT film inside.

**Figure 17:**
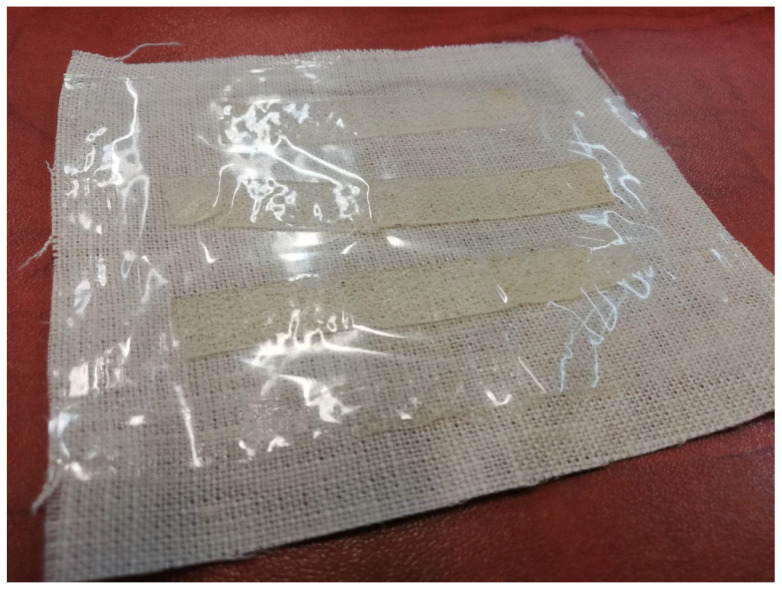
Bag covered with fabric on one side.

**Figure 18:**
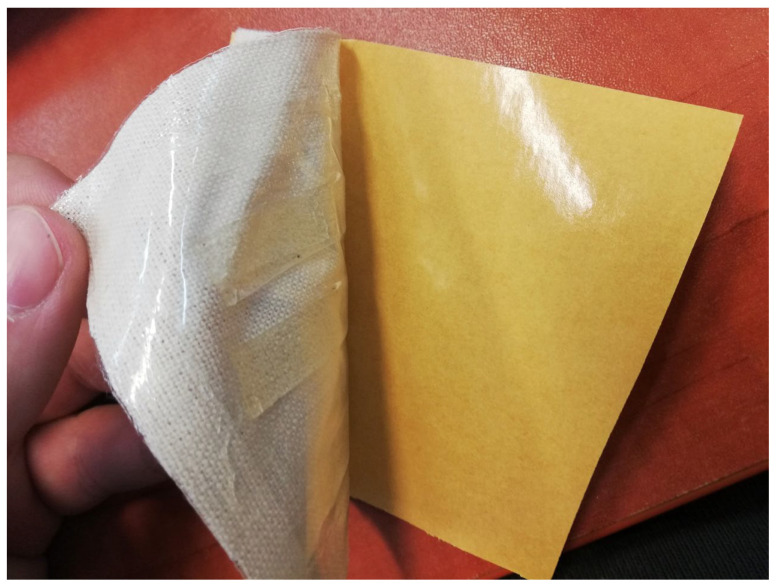
Prototype of dressing.

The system CMS/CMC/MMT-Na was selected for wound dressing model preparation. The biocomposite film was dried in a desiccator and then sealed in a foil bag. One side of this bag was permeable (perforated) (Figure 16). Then, the bag was covered with fabric on the impermeable side to create a protective layer for the patch and, at the same time, allow it to easily stick to the edges of the wound Figure 17. The whole dressing was protected with adhesive paper (Figure 18).

## 4. Conclusions

The film CMS/CMC containing 5 pph of MMT-Na was selected as the optimal material for the preparation of wound dressings with a high absorption capacity.

This choice can be justified as follows. The idea of using the developed film for superficial wounds with exudate primarily requires absorbent dressing; hence, parameters such as swelling and moisture absorption were crucial. At the same time, the low solubility of the film was important. Here, the two-component CMS/CMC film showed better parameters. The use of the MMT-Na filler allowed for the improvement of these parameters, and at the same time, had an impact on mechanical strength. Addition of the filler increased water binding and absorption capacity, and also increased the tensile strength of the film, making the dressing more durable and universal. The proposed construction of the absorbent dressing is only one of the possible options because the increased strength of the film, thanks to the filler, allows it to be used in other dressing systems as well.

## Data Availability

The raw/processed data required to reproduce these findings cannot be shared at this time as the data is part of an ongoing study.
